# Exome Sequencing of a Multigenerational Human Pedigree

**DOI:** 10.1371/journal.pone.0008232

**Published:** 2009-12-14

**Authors:** Dale Hedges, Dan Burges, Eric Powell, Cherylyn Almonte, Jia Huang, Stuart Young, Benjamin Boese, Mike Schmidt, Margaret A. Pericak-Vance, Eden Martin, Xinmin Zhang, Timothy T. Harkins, Stephan Züchner

**Affiliations:** 1 John P. Hussman Institute for Human Genomics, University of Miami Miller School of Medicine, Miami, Florida, United States of America; 2 Roche Diagnostics Corporation Inc., Indianapolis, Indiana, United States of America; 3 Center for Computational Sciences, University of Miami Miller School of Medicine, Miami, Florida, United States of America; Louisiana State University, United States of America

## Abstract

Over the next few years, the efficient use of next-generation sequencing (NGS) in human genetics research will depend heavily upon the effective mechanisms for the selective enrichment of genomic regions of interest. Recently, comprehensive exome capture arrays have become available for targeting approximately 33 Mb or ∼180,000 coding exons across the human genome. Selective genomic enrichment of the human exome offers an attractive option for new experimental designs aiming to quickly identify potential disease-associated genetic variants, especially in family-based studies. We have evaluated a 2.1 M feature human exome capture array on eight individuals from a three-generation family pedigree. We were able to cover up to 98% of the targeted bases at a long-read sequence read depth of ≥3, 86% at a read depth of ≥10, and over 50% of all targets were covered with ≥20 reads. We identified up to 14,284 SNPs and small indels per individual exome, with up to 1,679 of these representing putative novel polymorphisms. Applying the conservative genotype calling approach HCDiff, the average rate of detection of a variant allele based on Illumina 1 M BeadChips genotypes was 95.2% at ≥10x sequence. Further, we propose an advantageous genotype calling strategy for low covered targets that empirically determines cut-off thresholds at a given coverage depth based on existing genotype data. Application of this method was able to detect >99% of SNPs covered ≥8x. Our results offer guidance for “real-world” applications in human genetics and provide further evidence that microarray-based exome capture is an efficient and reliable method to enrich for chromosomal regions of interest in next-generation sequencing experiments.

## Introduction

Despite the continued increases in next-generation sequencing (NGS) platform throughput, the cost of obtaining and analyzing full genome sequences on a large number of human individuals remains prohibitive. Therefore, at least for the near future, large scale human genetic studies will rely on techniques that select and enrich for chromosomal regions of interest prior to sequencing. This approach will allow for the efficient evaluation of the hundreds of individual samples typically required to detect risk-associated genetic variation in common complex disorders. In addition to the adaptation of PCR and microfluidics-based techniques to select genomic regions of interest [1], [2], new versions of array-based and solution-based hybridization methods provide promising genomic enrichment approaches [3]–[9]. Recently, Roche Nimblegen has made available a microarray-based sequence capture system that targets the majority of coding exons as listed in the Consensus Coding Sequences (CCDS) collection. This human exome capture array covers 33 Mb of genomic sequence, comprising ∼180,000 exons and over 500 miRNA genes. Since captured DNA fragments have an average size of 500 bp, NGS sequence reads frequently extend beyond targeted coding exons into the intron/exon boundary.

Although sequence variants in non-coding regulatory loci clearly have potential to result in pathology, Mendelian disease studies suggest that a large portion of disease-associated variation lies within coding exons [10], [11]. Recent studies seeking to identify clusters of rare variants related to complex phenotypes have also focused on coding exons [12]–[15], in part because available *in silico* tools allow for rapid assessment of the potential functional consequences of any novel variants.

While the sequences of five exomes have been examined in the context of full human genome sequences [16]–[20], to date 13 exome-scale sequences derived from capture methods followed by NGS sequencing have been described [3], [21]. These, along with smaller-scale studies, [6], [22] have carefully examined the efficiency, reproducibility, and uniformity of targeted genomic capture/enrichment using array-based methodologies, as well as the extent to which allelic bias and GC-content influence results.

Here, we present the results from eight complete exomes captured via 2.1 M feature capture arrays (Roche). NGS was carried out on the 454 Genome Sequencer FLX (GS FLX) platform (Roche). Eight individuals were selected from a three generation pedigree. This approach allowed for cross-validation of segregating variants and facilitated genotype evaluation. The samples used in this study were extracted from peripheral blood, and DNA was stored for 13 years, representing conditions that are not uncommon for larger human genetic disease sample collections. We outline an empirical optimization approach to genotype calling in individual exomes, assess the reliability of genotype calling, and provide an overall evaluation of array-based exome capture followed by NGS analysis on the 454 platform.

## Methods

### Human Subjects and DNA Samples

**Figure 1:**
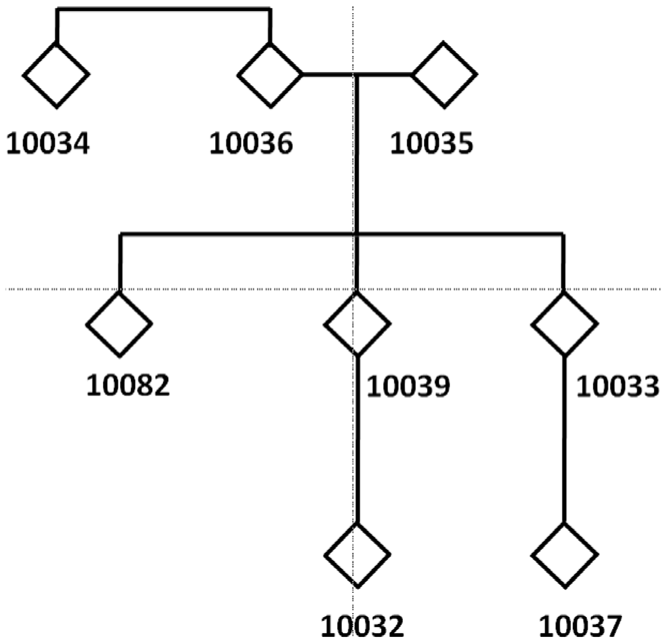
Studied three-generational pedigree. Pedigree of eight individuals of European descent that was studied with exome capture arrays.

We examined eight individuals from a three generation family pedigree (Fig. 1). All family members are of self-reported European descent. Written informed consent for genetic studies was obtained prior to initiating this study in agreement with protocols approved by the institutional review board (IRB) at the University of Miami Miller School of Medicine. DNA was extracted from peripheral blood leukocytes using automated DNA extraction. DNA samples were stored for approximately 13 years at the Biorepository of the John P. Hussman Institute for Human Genomics, University of Miami. Before usage in this study, DNA aliquots were re-precipitated and treated with RNAse to remove proteins and RNA remnants (Fig. S1).

### Exome Capture

Exome capture was performed using 5 ug of input DNA according to the manufacturer's protocol (Roche Nimblegen). Briefly, genomic DNA was nebulized for 1 minute using 45psi of pressure. Sheared DNA fragments were subsequently cleaned with the DNA Clean & Concentrator-25 Kit (Zymo Research) and a fragment size distribution ranging from 300 bp to 500 bp was verified via Bioanalyzer (Agilent). After end-polishing of the genomic fragments, the GS FLX Titanium adaptors were ligated to the sheared genomic fragments. Ligated fragments were next hybridized to the 2.1 M exome array within Maui hybridization stations, followed by washing and elution of array-bound fragmentsfrom the arrays within elution chambers (Nimblegen). Captured fragments were next subjected to 27 rounds of PCR amplification using primers targeting the Nimblegen linkers. Following elution, the capture efficiency was evaluated via q-PCR reactions. For additional details, see manufacturer's protocol that was modified for the human exome arrays (http://www.nimblegen.com/products/lit/SeqCap_UserGuide_Tit_Del_v1p0.pdf).

### Next-Generation Sequencing

Captured DNA samples were subjected to standard sample preparation procedures for 454 GS FLX sequencing as recommended by the manufacturer (Roche Inc.). Two full 454 GS FLX (Titanium) runs were performed for each of the eight samples with the exception of individual 10039, which was sequenced using a total of four full 454 GS FLX runs.

### Array-Based Genotyping

We processed all individuals on the Illumina 1 M Duo BeadChip, following the manufacturer's recommendations (Illumina Inc.). BeadChip arrays were scanned on the iScan instrument, and preliminary analysis was conducted using Illumina BeadStudio v3.29 software. Quality-control tests for genotyping calls included the following measures: samples were required to have an overall genotype call efficiency of ≥0.98; Mendelian inconsistency checks were performed using WASP [23]; reported gender and genetically determined gender were examined with the use of X-linked SNPs, and we required a conservative gencall score of 0.25. Variant loci with more than 10% missing genotypes and SNPs with minor allele frequency <1% across all individuals were dropped from the analysis. To determine high quality SNPs we applied the PLINK software [24].

### Capillary Sequencing

For follow-up confirmation of identified novel variants we also applied capillary sequencing. PCR primers were designed flanking approximately 200 bp of a given variant and sequenced on an ABI 3730 capillary sequencing instrument following standard procedures (Life Technologies). Capillary sequence reads were analyzed using the Sequencher software package (GeneCodes Inc.).

### Bioinformatics and Statistical Analyses

Next-generation sequencing data were initially processed using the GSMapper software package (Roche Inc.) supplied with the GS FLX instrument. High quality sequencing reads were aligned to the human genome reference sequence NCBI 36.1. Variants with respect to NCBI 36.1 reference sequence were identified with the GSMapper software (AllDiff and HCDiff reports). The AllDiff (all differences) strategy (output in the GSMapper AllDiff file) requires the following criteria for a variant to be reported: (1) At least two reads differ either from the reference sequence or from other reads aligned at a specific location. (2) there must be at least two non-duplicate reads that a) show the difference, b) have at least 5 bases on both sides of the difference, and c) have few other isolated sequence differences in the read. HCDiff (high confidence differences) requires the following criteria for a variant to be reported: 1) There must be at least 3 reads with the difference.; 2) There must be both forward and reverse reads showing the difference, unless there are at least 5 reads with quality scores over 20 (or 30 if the difference involves a 5-mer or higher).; 3) If the difference is a single-base overcall or undercall, then the reads with the difference must form the consensus of the sequenced reads. Coverage depth at all detected variants and at all positions corresponding to an Illumina 1 M genotype were extracted for the purpose of comparison with the output files HCDiff and AllDiff generated by the Roche GeneMapper software.

### Empirical Optimization of Genotype Calling

To determine empirical allele frequency threshold values for assigning sequence-derived genotypes in our study, we obtained genotype data for each individual in our study from a set of 44,513 Illumina 1 M Duo SNPs that fell within our targeted exonic regions. The data set was arbitrarily divided into two approximately equal SNP sets. We used the first set as a training set to establish calling thresholds, and the second set was used for the validation of the calling strategy. We defined lower (cl) and upper (cu) thresholds such that if the percent of non-reference reads is less than cl, then the genotype is called homozygous for the reference allele; if the percent of non-reference reads was between cl and cu the genotype is called heterozygous; and if the percent of variant reads was greater than cu the genotype is called homozygous for the non-reference allele. For each given depth of coverage, we determined the threshold values for assigning genotypes (based on the frequency of non-reference alleles present among the set of sequence fragments) such that the genotyping calls yielded the highest percent identity with the Illumina training data set. Due to the nature of NGS data, more training data were available for some call depths than others. We required a minimum of 50 genotypes be present in the training set to set a threshold at a given coverage depth. Using these criteria, we established calling thresholds for the range of 3X to 23X coverage.

## Results and Discussion

### Exome Capture

Prior to exome capture, all DNA samples underwent quality controls, including agarose gel runs and spectrophotometric quantification (Fig. S1). The capture/enrichment of targeted exons using the human exome array was carried out following the manufacturer's recommendations (see Methods). Successful enrichment was initially evaluated by four real-time qPCR control targets present on the array. After elution of the sequence capture reactions, an estimated enrichment of control targets ranging from 53 to 102-fold over background was measured based on qPCR data (Table S1).

### NGS Run Statistics

For each of eight individuals we obtained between 0.7 and 1.3 Gb of genomic sequence from two GS FLX runs. One subject (10039) was sequenced with a total of four GS FLX runs, resulting in ∼2 Gb of data. The average read length obtained was 340 bp (Table 1). Fragment sizes ranged from 50 bp (the minimal length used for analysis) to a maximum of 835 bp. All sequencing runs yielded comparable average sequence lengths and total sequence amounts. Run statistics for each sample are given in Table 1.

**Table 1:** NGS run statistics for eight exomes aligning high-quality sequencing reads.

Individual	mapped bases (bp)/ % of total bases	# mapped unique reads§/ % of total reads	Unique reads§in target region/ % of all reads	Target Base Coverage	Average read length (bp)	Max read length * (bp)
10032	926,438,032 (99.79%)	2459464 (99.35%)	1854613 (78.02%)	92.50%	369	677
10033	814,175,547 (99.73%)	2275083 (99.26%)	1570217 (71.60%)	91.20%	345	635
10034	750,594,870 (99.76%)	2169892 (99.33%)	1532537 (73.18%)	90.90%	335	732
10035	1,146,776,462 (99.69%)	3293074 (99.28%)	2457890 (77.45%)	93.60%	339	755
10036	1,333,018,529 (99.71%)	3995447 (99.21%)	3099809 (80.26%)	93.20%	328	728
10037	892,370,696 (99.75%)	2421459 (99.30%)	1875210 (80.15%)	92.80%	360	736
10039	912,259,209 (99.78%)	2583714 (99.29%)	1984028 (79.68%)	94.20%	347	755
10082	670,270,644 (99.63%)	2197618 (98.80%)	1753660 (82.92%)	92.20%	299	835
**Average**	**930,737,999(99.73%)**	**2,674,468 (99.23%)**	**2,015,995 (77.91%)**	**92.58%**	**340**	**732**

#Number of.

*The minimum read length required was 50 bp.

§ Before alignment, all raw reads were screened for duplicate reads, which are introduced by amplification steps during library preparation on next-generation platforms.

Only the first two runs are shown for better comparison.

### Target Coverage

**Figure 2:**
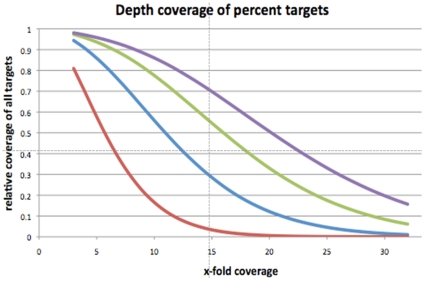
Sequence coverage of targeted exons. The graph illustrates the cumulative coverage of targeted bases after sequencing 0.5 Gbp (red), 1 Gbp (blue), 1.5 Gbp (green), and 2 Gbp (purple). 1 Gb resulted in nearly 10x coverage of 50% of all targets; 2 Gb of data increase this number to 88%. Depending on a studies goal, maximum coverage might not always be required.

Initial analysis of the NGS sequence results was conducted using the GSMapper software package, which is optimized for the long read alignment produced by the GS FLX platform. By applying default GSMapper settings, which exclude low quality reads from alignment, >99% of all remaining sequence fragments mapped to the human genome reference NCBI36.1 (Table 1). Between 71.6% and 82.9% of all reads from each individual fell within a region targeted by the human exome array, yielding an average capture efficiency of 77.9% (Table 1). For comparison, we would expect <2% of reads to uniquely map to targeted regions were no enrichment strategy employed. Our capture efficiencies resulted in 91% to 94% of all targeted bases being covered by at least three sequence reads (Fig. 2). On average, two full 454 FLX runs (or ∼1 Gb of sequence) resulted in ∼50% of all targets being covered by ten or more reads and ∼30% of all targets covered by a depth of greater than 15x (Fig. 2). The average coverage over all individuals was 8.82. In individual (10039), with 2 Gb of sequence data available, 98% of targets were covered at ≥3X, 86% of all targets were covered at ≥10X, and ∼70% of targets were covered ≥15x (Fig. 2).

### Variant Detection and Genotype Designation

Variant detection was performed using a combination of different sequence read stringencies with the GSMapper software, resulting in the generation of high confidence variant detection (HCDiff) and a less conservative approach to detect all possible sequence variants (AllDiff) (see Methods). Across all eight (related) individuals that were sequenced with two full 454 runs, we identified 21,533 unique variants, including SNPs and small insertions and deletions (indels). Per individual we found 6790 – 11,038 loci heterozygous or homozygous for a variant allele based on HCDiff; an average of 8744 (Table 2). Between 444 and 1163 variants per individual were not reported in dbSNP (v128) and were thus categorized as putative novel alleles. On average 297 putative novel non-synonymous alleles were identified per individual (Table 2). Individual 10036 yielded the most SNPs and individual 10034 showed the least variants of all samples (Table 2). As expected, this generally corresponds with the number of mapped reads covering targeted regions in these samples, where 10036 had the most (4.0 M) and 10034 the least number of reads (2.2 M) of all samples (Table 1). We note, however, that the greatest benefits for SNP detection from adding additional read data occurs as reads are increased in the range of 1 to approximately 2.6 M reads, with more modest detection increases observed as data is added beyond 2.6 M reads. We also searched for variants that occurred +/− 2 bp from an exon boundary as they have a high potential to interfere with exon/intron splicing activity. We identified on average 54 such variants per individual (Table S2). Individual 10039 was sequenced with two additional GS FLX runs, yielding nearly twice the number of sequencing reads and increased target coverage. In 10039 we identified 14,284 variants (88% known to dbSNP) with 624 of them being novel non-synonymous SNPs (Table S3).

**Table 2:** Genomic variants detected in eight exomes based on 2 454 GS FLX runs of aligned data.

Individual	10032	10033	10034	10035	10036	10037	10039	10082	Avg.	Range
**KNOWN VARIANTS**	**7962**	**6342**	**6346**	**9480**	**9875**	**7924**	**9398**	**7165**	**8062**	**6342–9875**
Non-Synonymous	3467	2687	2749	4059	4257	3363	3952	3108	3455	2687–4257
indel	49	38	34	69	73	41	65	56	53	34–73
SNP	3418	2649	2715	3990	4184	3322	3887	3052	3402	2649–4184
Synonymous	4495	3655	3597	5421	5618	4561	5446	4057	4606	3597–5618
indel	19	20	19	38	35	29	28	30	27	19–38
SNP	4476	3635	3578	5383	5583	4532	5418	4027	4579	3578–5583
**NOVEL VARIANTS**	**607**	**456**	**444**	**844**	**1163**	**610**	**748**	**591**	**683**	**444–1163**
Non-Synonymous	344	254	244	486	723	347	402	337	392	244–723
indel	49	44	31	118	296	48	58	115	95	31–296
SNP	295	210	213	368	427	299	344	222	297	210–427
Synonymous	263	202	200	358	440	263	346	254	291	200–440
indel	21	16	19	44	76	21	29	31	32	16–76
SNP	242	186	181	314	364	242	317	223	259	181–364
**Total**	**8569**	**6798**	**6790**	**10324**	**11038**	**8534**	**10146**	**7756**	**8744**	**6790–11038**

In order to take advantage of having sequenced related individuals within a known pedigree structure, we also combined raw sequencing read data from three siblings (10082, 10033, 10039) and analyzed them as a quasi single individual. As these individuals share parental origin, we hoped to detect additional variants above calling thresholds at regions of low coverage. Indeed we were able to identify 17,498 variants based on HCDiff in the combined dataset compared to 15,545 unique variants in the original HCDiff files. Thus, the detection of an additional 11% of variants renders this approach an attractive strategy for maximizing variant detection. A possible application would be the combination of data from multiple affected individuals within a single pedigree in order to maximize the probability of sampling a risk-associated allele in the context of low overall sequence coverage. In this case, even though the actual risk locus may receive inadequate coverage within any given affected individual, the pooling of reads at the locus across a group of such individuals would greatly increase the probability of the mutant allele being detected.

### Sensitivity of Variant Identification

**Figure 3:**
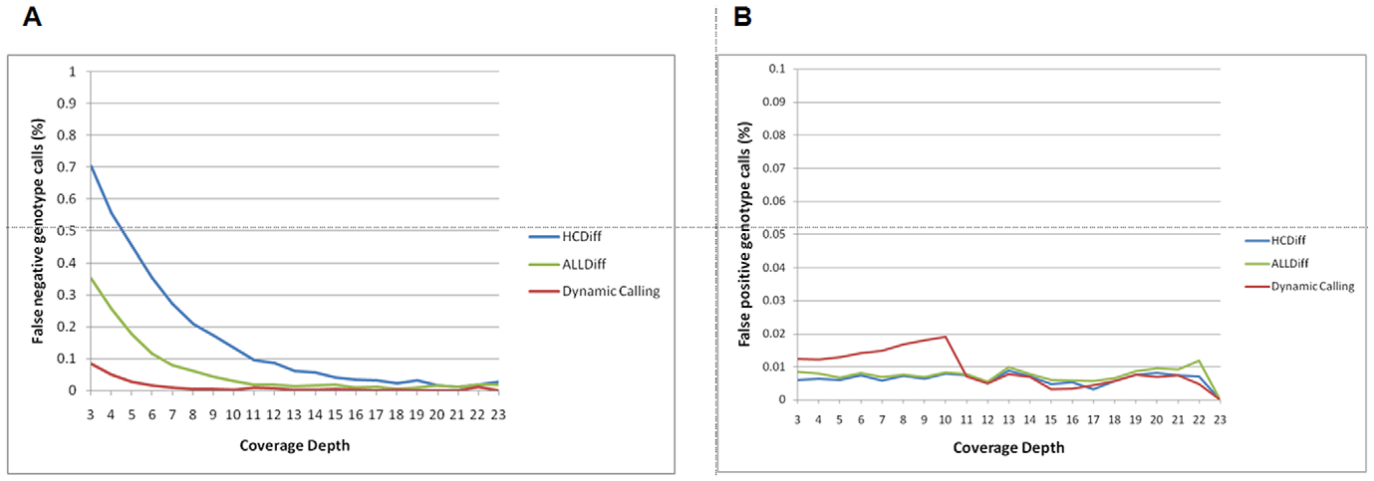
Estimated error rates. Sensitivity of genotype calling based on HCDiff SNPs, AllDiff SNPs, and the proposed coverage-dependent genotype calling approach. A) False negative rates are based on concordance with a subset of 44,513 SNPs that overlapped with genotypes obtained with Illumina 1 M Duo BeadChips. The coverage-dependent variant calling approach that calibrates cut-off rates according to array-based genotypes is the most sensitive method, detecting >96% of SNPs at 5x coverage and >99% of all SNPs at ≥8x coverage. B) False positive rates. HCDiff is the most conservative algorithm, resulting in a smaller false positive rate, while the more relaxed dynamic genotype calling algorithm results in twice as high error rates at lower coverage.

Ideally, error estimates for genotype calling should be based on independently obtained and validated sequence data. In lieu of the availability of known exome sequences for our eight individuals, we genotyped all eight samples with the Illumina 1 M Duo BeadChip, which contains ∼1×10^6^ SNP markers. We applied a number of stringent quality checks to all genotype calls derived from these arrays to define a subset of high quality SNPs (see Methods). Only genotypes that overlapped with targeted exons were retained for further analysis. This resulted in 44,513 high quality SNP loci for comparison to NGS data, which we used to calculate sensitivity levels. Here, we define false negative as the frequency at which individuals heterozygous or homozygous for the variant alleles are incorrectly called as homozygous for the reference allele. This relaxed definition of false negatives recognizes that many exome capture studies will have genetic variant discovery as a primary goal. When using the two different outputs from GSMapper, ALLDiff and HCDiff, compared to the Illumina BeadChip genotypes, a range is observed for the false negative rates. Applying more inclusive AllDiff criteria, the false negative rate was 6% at 8x and 1.8% at 15x coverage. When using the very conservative HCDiff criteria a false negative rate of 21% at 8x and 4% at 15x was calculated (Fig. 3). A potentially more practical approach to calculating false negative rates is the cumulative false negative rate, which considers all targets covered at a given depth and higher. Under this assumption, targets covered ≥8x were incorrectly called reference in 6.7% of all tested SNPs for HCDiff and in 2% for ALLDiff.

### Empirical Coverage-Based Variant Assessment

**Figure 4:**
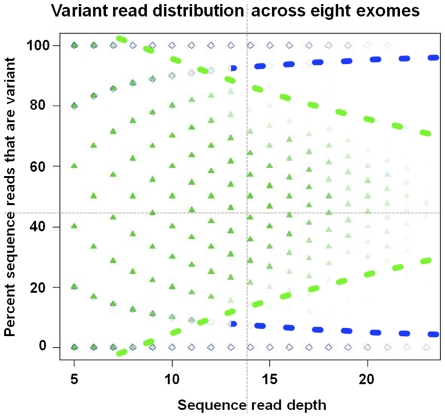
Variant read distribution across eight exomes. Illustration of the dynamic nature of optimal cut-off rates for calling heterozygous/homozygous variants. At lower coverage (<10x) the ideal cut-off is 88% variant reads in our data, while it is 78% at coverage ≥20. Optimal usage of data should take advantage even of low covered targets. Data are based on comparison to Illumina genotyped SNPs. Green triangles: Illumina heterozygous genotypes, Blue diamonds: Illumina homozygous genotypes. NGS genotypes are placed according to their percent variant reads (y axis).

A significant parameter that must be determined when assigning genotypes based on NGS data is the cut-off proportion of variant allele reads for a genotype to be called heterozygous or homozygous. Several end-user software packages (e.g. Lasergene, CLC) allow for a static, but adjustable, cut-off threshold across all covered nucleotide positions. More sophisticated strategies have been developed, relying on maximum likelihood (e.g.,[26]) and prior knowledge of allele frequencies ([27]), and these have been implemented to target data from short-read sequencing platforms. To empirically determine genotype-calling thresholds on a coverage dependent basis we used Illumina genotype data to optimize our genotype calling cut-offs (and thereby variant detection). Rather than applying a rigid cut-off rate for heterozygous variant reads across all loci (e.g. >30% variant reads), the optimal calling threshold was defined as that frequency which maximizes the number of correct NGS genotype calls (based on comparison to Illumina genotyping data). The resulting thresholds were determined for each discrete coverage depth (3x, 4x, etc) (Fig. 4). As described below, this empirically determined variant calling approach improved the overall sensitivity to detect variants compared to the standard HCDiff variant detection algorithm, particularly at low coverage targets (Fig. 3A). Typical cut-off values produced by our method were cl = 12% and cu = 88% at lower coverage depth and increased to cl = 22% and cu = 78% at coverage ≥15 (Fig. 4). Applying this approach, we were able to identify at 5x coverage more than 96% of all heterozygous and homozygous changes reported by the Illumina 1 M BeadChip (Fig. 3A). At 8x coverage 99.99% of SNPs were identified (Fig. 3A). Although this approach allows us to maximize sensitivity, the rate of erroneously reported variants (specificity) is modestly increased at coverage depths of ≤10x (Fig. 3B).

The rationale for this genotype calling approach is derived from the fact that errors may result from a combination of array-capture methodology, sequencing technology, as well as from sampling error at loci. There are a number of difficulties involved in disentangling these factors. Normal variation in hybridization conditions and sequencing runs will likely cause the relative contribution of each of these sources of error to fluctuate between individual samples and sequencing runs. Furthermore, aspects of the sequenced population itself (e.g. heterozygosity levels) may also influence the overall performance of genotype calling strategies. The empirical optimization method proposed here allows all these factors to be taken into account without having to independently assess them. Given the fairly large and representative group of SNPs evaluated, we expect that this basic approach for generating cut-off values will generally be valid for other, unrelated samples, but the exact values will likely prove contingent upon the specific enrichment and sequencing technologies applied.

### Specificity of Variant Identification

Here, we restrict our definition of false positives to the appearance of an allele at a sequenced locus that is not present in the Illumina dataset for the individual. This is a conservative approach as it is likely over-estimating error; differences between Illumina genotyping data and NGS data, when independently assessed with capillary sequencing [28], are often resolved in favor of NGS. We used 112,384 genotypes from the Illumina 1 M to examine specificity across the targeted exome, regardless of whether they were present in any of the eight individuals. Of the three tested approaches, the HCDiff algorithm produced the lowest false positive rate of 0.7% (Fig. 3B). ALLDiff resulted in an only marginally higher false positive rate. As expected, the empirical coverage-based genotype calling approach had decreased specificity at lower coverage; the average rate of artifactual variant calls was 1.5% at targets covered with 3 to 11 reads. For studies that are willing to accept this rate of specificity, it appears to be worth considering a more aggressive genotype calling approach at regions of low coverage. At sequence read coverage >15x, the differences in error rates between the different variant calling approaches become negligible.

As another measure of the incidence of false discovery, we also attempted to confirm 53 randomly chosen HCDiff SNPs across the exome by capillary sequencing. We found that all 53 SNPs were correctly called as heterozygous and homozygous variants, thereby verifying the low rate of incorrect genotypes obtained from the HCDiff algorithm.

### Mendelian Inheritance Checks

Finally, we calculated the total number of Mendelian inconsistencies in the pedigree resulting from NGS genotype calls using the HCDiff SNPs. These errors could either be attributed to improper genotype assignment or to authentic de-novo events. As summarized in Table S4, the Mendelian error is 0.5% at 10x and 0.2% at 15x. As expected, the frequency of Mendelian errors diminishes as a function of increased sequence depth. This indicates that a significant component of Mendelian inconsistencies were genotyping errors attributable to sampling error resulting from insufficient sequencing coverage. The contribution of de novo events to Mendelian inconsistencies appears low, as we were not able to confirm any *de novo* mutation in 47 suspected variants (see below). Therefore, when lacking available genotyping data to estimate sensitivity and specificity, Mendelian inconsistencies derived from available pedigree structures can assist in gauging overall levels of genotype error and could aid in the optimization of genotype calling algorithms.

### De-Novo Variation

We made use of the available pedigree to search for evidence of de-novo changes. By filtering the entirety of detected variants (Table 2) for novel variants that occurred in an offspring (10033), but not in parents (10035 and 10036), we identified 47 changes suggestive of de-novo mutation events. However, subsequent capillary sequencing confirmed none of these variants as true de-novo events. While this initially suggests a disturbingly high error rate, it must be considered that by selecting alleles that appear to be de novo mutants, we are also strongly biasing towards the set of artifactual SNP calls in the offspring as well as those SNPs we failed to detect in either parents. Both of these latter categories will appear identical to de novo events in the pedigree data set. For comparison, when loci were randomly selected for capillary sequence validation, we observed dramatically lower levels of error (see above). Nevertheless, the failure to authenticate any of the putative de novo mutations does imply a relatively low de-novo mutation rate in the exome. If one extrapolates from Nachman et al. [25], the de-novo mutation rate in the exome should be no more than two variants per generation (1% of 175 de-novo mutation events per generation genome-wide), and thus close to the false positive error rate of the sequencing approach described here (Fig. 3B). In addition, de novo mutations in coding exons likely carry an increased risk for compromising organismal viability, as compared to mutations elsewhere in the genome, and hence they are less likely to be sampled among successful births.

### Conclusion

Genomic enrichment methods and NGS platforms are currently undergoing rapid development, leading to leap frog advancements in genomic discovery tools. The results described here can provide only a snapshot in time of the possibilities and limitations of a combined array-based hybridization and NGS strategy. Our data indicate that exome scale array capture enrichment provides a powerful tool for genomic targeting and variant detection in NGS studies.

With the present approach we have achieved ≥8x coverage of ∼90% of all targets with ∼2 Gb of aligned sequence reads (Fig. 2). Our coverage-dependent sensitivity rates suggest that between 80% and 99.99% of all variants (depending on what strategy is used) will be correctly identified at this coverage level (Fig. 3). We have detected up to 13,605 SNPs per individual based on the conservative HCDiff algorithm (Table S3). Taking into account a sensitivity of 84% with HCDiff (including all targets covered ≥3 reads) (Fig. 3A), we extrapolate a full set of 15,781 SNPs in individual 10039; this is comparable to the SNP numbers recently reported for six Caucasian exomes [29]. This also underlines, however, that near complete variant detection requires extensive sequence coverage in order to overcome variability in coverage depth across target regions.

Since NGS performance is correlated to coverage depth, it is of key interest for investigators to assess the “right” amount of sequencing in order to maximize respective study goals within budgetary constraints. Our data should provide empirical guidelines for such decisions, which are likely to be generalizable when using the same technologies (Figs. 2 and 3). Although the safest strategy appears to be an average coverage ≥20x, a number of targets will be covered with less sequencing reads in any study due to enrichment uniformity issues with hybridization-based approaches. For at least the next few years, studies involving hundreds of individuals will find the expense of obtaining optimal high coverage across all target loci a daunting prospect. Partly to address such challenges, we have proposed an empirical coverage-based genotype calling approach. Using this approach we were able to greatly improve detection of variant alleles at 3x - 15x coverage (Fig. 3A). The “cost” of false positives (i.e. reduced specificity), although more than twice that of conservative algorithms, was below 1.5% and might be acceptable for rare variant discovery studies (Fig. 3B). More conservative calling approaches will be preferable for other study designs.

In summary, the human exome capture array combined with GS FLX sequencing provides a powerful means to detect genomic variation in >90% of all human exons. The results and models presented here should aid future study designs aiming at detection of exonic sequence variation.

## Supporting Information

Figure S1 Spectrophotometric measurements (A) and agarose gel runs (B) of DNA samples prior to sequence capture were part of the quality assessment of the eight DNA aliquots.
(2.94 MB TIF)

Table S1 (0.03 MB DOC)

Table S2 (0.03 MB DOC)

Table S3 (0.03 MB DOC)

Table S4 (0.03 MB DOC)
