# Correction: Exome Sequencing of a Multigenerational Human Pedigree

**DOI:** 10.1371/annotation/b0fe9dd5-16e1-4b50-b590-263518fbd5eb

**Published:** 2009-12-28

**Authors:** Dale J. Hedges, Dan Burges, Eric Powell, Cherylyn Almonte, Jia Huang, Stuart Young, Benjamin Boese, Mike Schmidt, Margaret A. Pericak-Vance, Eden Martin, Xinmin Zhang, Timothy T. Harkins, Stephan Züchner

The first author's name is not spelled correctly. It should be: Dale J. Hedges. The correct citation is: Hedges DJ, Burges D, Powell E, Almonte C, Huang J, et al. (2009) Exome Sequencing of a Multigenerational Human Pedigree. PLoS ONE 4(12): e8232. doi:10.1371/journal.pone.0008232 

