# Supplementary figures and images for: Exome Sequencing of a Multigenerational Human Pedigree
Source: PLoS One. 2009 Dec 14;4(12):e8232. doi: 10.1371/journal.pone.0008232 (PMC2788131; doi:10.1371/journal.pone.0008232)

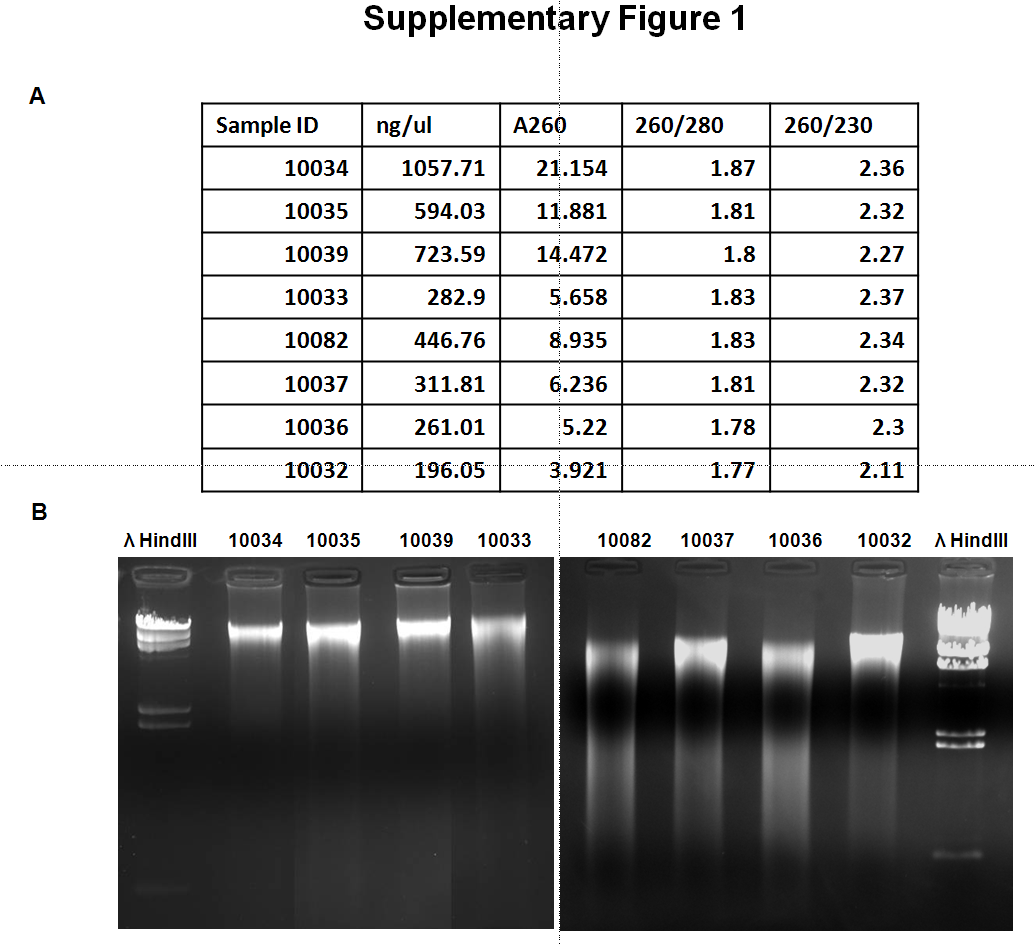

Supplement: Figure S1 — Spectrophotometric measurements (A) and agarose gel runs (B) of DNA samples prior to sequence capture were part of the quality assessment of the eight DNA aliquots. (2.94 MB TIF) [file pone.0008232.s001.tif]
