# Supplementary material for: Exome Sequencing of a Multigenerational Human Pedigree
Source: PLoS One. 2009 Dec 14;4(12):e8232. doi: 10.1371/journal.pone.0008232 (PMC2788131; doi:10.1371/journal.pone.0008232)
Supplement: Table S1 — (0.03 MB DOC) [file pone.0008232.s002.doc]

| **Sample** | **Fold Enrichment Estimate by qPCR*** | **Total Yield (ug)** |
| --- | --- | --- |
| 10032 | 91.3 | 28.7 |
| 10033 | 52.7 | 14.4 |
| 10034 | 61.8 | 13 |
| 10035 | 72.0 | 13 |
| 10036 | 102.8 | 14 |
| 10037 | 57.6 | 10.8 |
| 10082 | 73.7 | 7.9 |
| 10039 | 64.8 | 12 |
| PCC (Process Contamination Control) | PASS | n.a. |
| NTC (No Template Control) | PASS | n.a. |
| 5 ng genomic DNA (Positive Amplification Control) | PASS | n.a. |

**Supplementary Table S1.** Enrichment results for eight exome samples.

* “Enrichment” refers to the average of four internal target controls that were amplified with qPCR before and after capture/ enrichment to generally judge whether the hybridization generally worked as expected.
