# Supplementary material for: Exome Sequencing of a Multigenerational Human Pedigree
Source: PLoS One. 2009 Dec 14;4(12):e8232. doi: 10.1371/journal.pone.0008232 (PMC2788131; doi:10.1371/journal.pone.0008232)
Supplement: Table S2 — (0.03 MB DOC) [file pone.0008232.s003.doc]

**Supplementary Table S2.** Number of detected variants near splice junctions.

| **Individuals** | **Count of SNPs +/- 2bp of a splice junction** |
| --- | --- |
| 10032 | 57 |
| 10033 | 42 |
| 10034 | 45 |
| 10035 | 66 |
| 10036 | 67 |
| 10037 | 52 |
| 10039 | 59 |
| 10082 | 44 |
