# Supplementary material for: Exome Sequencing of a Multigenerational Human Pedigree
Source: PLoS One. 2009 Dec 14;4(12):e8232. doi: 10.1371/journal.pone.0008232 (PMC2788131; doi:10.1371/journal.pone.0008232)
Supplement: Table S3 — (0.03 MB DOC) [file pone.0008232.s004.doc]

**Supplementary Table S3.** Variants identified in individual 10039 based on four 454 FLX sequencing runs.

| **10039** | |
| --- | --- |
| **KNOWN VARIANTS** | **12605** |
| Non-Synonymous | 5328 |
| indel | 109 |
| SNP | 5219 |
| Synonymous | 7277 |
| indel | 45 |
| SNP | 7232 |
| **NOVEL VARIANTS** | **1679** |
| Non-Synonymous | 1050 |
| indel | 426 |
| SNP | 624 |
| Synonymous | 629 |
| indel | 99 |
| SNP | 530 |
| **Total** | **14284** |
