# Supplementary material for: Exome Sequencing of a Multigenerational Human Pedigree
Source: PLoS One. 2009 Dec 14;4(12):e8232. doi: 10.1371/journal.pone.0008232 (PMC2788131; doi:10.1371/journal.pone.0008232)
Supplement: Table S4 — (0.03 MB DOC) [file pone.0008232.s005.doc]

**Supplementary Table S4.** Mendelian error rates at 5x, 10x, and 15x coverage.

| **Coverage** | **Errors** | **Markers** | **Error Rate** |
| --- | --- | --- | --- |
| ≥5x | 322 | 17702 | 0.01819 |
| ≥10x | 25 | 5156 | 0.004849 |
| ≥15x | 1 | 471 | 0.002123 |
